# Resource Utilization and Throughput in Pediatric Abdominal Pain among Attendings, Residents, and Advanced Practice Clinicians

**DOI:** 10.5811/westjem.43593

**Published:** 2025-11-18

**Authors:** AG Nuwan Perera, Robert Tisherman, Raymond Pitetti, Kavitha Conti, Samantha A. Ohl, Jennifer Dunnick

**Affiliations:** * University of Pittsburgh School of Medicine, Department of Pediatrics, Division of Pediatric Emergency Medicine, Pittsburgh, Pennsylvania; † Duke University School of Medicine, Department of Orthopedic Surgery, Division of Sports Medicine, Durham, North Carolina; ‡ Duke University School of Medicine, Department of Pediatrics, Division of Pediatric Emergency Medicine, Durham, North Carolina

## Abstract

**Introduction:**

Our goal was to assess the impact of emergency department (ED) clinician category on length of stay (LOS) and resource utilization in children presenting with abdominal pain.

**Methods:**

We conducted a retrospective chart review of all subjects 4–18 years of age at a quaternary-care pediatric ED between May 2021–April 2022 presenting with a chief complaint of abdominal pain. Collected data included demographics, LOS, disposition, 72-hour return visits, lab tests and radiology studies, consults, and emergency clinician category. We defined clinician categories as attending only, advanced practice clinician (APC) only, or supervised resident encounters. Medically complex and high-acuity cases were excluded. We performed statistical comparisons with ANOVA, chi-squared, and Kruskall-Wallis tests. Binomial logistic regression addressed the effects of the covariates age, sex, race, and acuity level.

**Results:**

We included 3,874 episodes. Of these, 622 (16%) visits were seen by an attending only, 1,018 (26%) by APCs, and 2,234 (58%) by supervised residents. Controlling for covariates, the average APC encounter lasted 17 minutes longer than the average attending encounter (293 minutes vs 276 minutes, *P* < .005, 95% CI −29.9, −4.0) and 21 minutes longer than the average resident encounter (293 minutes vs 272 minutes, *P* <.001, 95% CI 11.4–30.6). There were no significant differences in admission rates (attending: 128/622 [20.6%]; APC: 226/1,018 [22.2%]; resident: 477/2,234 [21.4%]; *P* = .63), or 72-hour return rates (attending: 30/622 [4.8%]; APC: 41/1,018 [4.0%]; resident: 99/2,234 [4.4%]; *P* = .61). Compared to attending-only encounters, APC encounters were more likely to include a consult (127/622 [20.4%] vs 292/1,018 [28.7%]; adjusted odds ratio (aOR) 1.51, 95% CI 1.18–1.93); less likely to include a computed tomography (CT) (13/622 [2.1%] vs 7/1,018 [0.7%]; aOR 0.31, 95% CI 0.12–0.79); more likely to include a radiology study (484/622 [77.8%] vs 873/1,018 [85.8%], aOR 1.64, 95% CI 1.26–2.14); and more likely to include lab testing (329/622 [52.9%] vs 669/1,018 [65.7%], aOR 1.62, 95% CI 1.30––2.00). Compared to supervised resident encounters, APC encounters were more likely to include a consult (518/2,234 [23.2%] vs 292/1,018 [28.7%], aOR 1.35, 95% CI 1.14–1.61); less likely to include a CT (36/2,234 [1.6%] vs 7/1,018 [0.7%], aOR 0.43, 95% CI 0.19–0.98); more likely to include a radiology study (1603/2,234 [71.8%] vs 873/1,018 [85.8%], aOR 2.41, 95% CI 1.97–2.96); and more likely to include lab testing (1,230/2,234 [55.1%] vs 669/1,018 [65.7%], aOR 1.63, 95% CI 1.39–1.92). Attending-only encounters were more likely to include radiology studies compared to resident encounters (484/622 [77.8%] vs 1,603/2,234 [71.8%], aOR 1.47, 95% CI 1.18–1.83), but they were otherwise similar in diagnostic utilization.

**Conclusion:**

In our study of pediatric patients with abdominal pain, APC encounters had longer length of stay and were more likely to include lab testing, radiology studies, and consults than resident or attending-only encounters. This suggests that emergency clinician category may be associated with resource utilization, and further research could help optimize healthcare utilization.

## INTRODUCTION

Emergency department (ED) crowding is a significant health system concern that contributes to increased cost, decreased access, and unfavorable outcomes in pediatric health care.^1^,^2^ While ED crowding is multifactorial, one important factor is ED throughput, defined as the time spent in the ED by a patient, also known as length of stay (LOS).^3^ The ED throughput itself is impacted by a variety of factors, including resource utilization and clinician staffing in the ED.

One response to physician staffing shortages has been to increase the use of advanced practice clinicians (APC) such as nurse practitioners (NP) and physician assistants (PA). Research involving general EDs has shown many benefits to adopting this strategy, including decreasing staffing costs and waiting room times without resulting in negative effects on general ED flow, clinical safety, or patient experience.^4^,^5^ An area of growing research in pediatrics is the effect of the ED clinician category on throughput and resource utilization. In one pediatric study, low-acuity pediatric patients evaluated by PAs in the ED had a statistically, but not clinically significant, longer ED LOS when compared to children seen by pediatricians.^6^

Pediatric research has also started investigating resource utilization by chief complaint. A 2021 study on bronchiolitis found that NPs were less likely to order tests or treatments.^7^ Variability in resource utilization for high-acuity and diagnostically complex chief complaints, such as abdominal pain, have not yet been studied in pediatric patients. Up to 10% of all pediatric ED visits are for complaints of abdominal pain, with diagnoses varying from benign conditions like constipation to urgent ones such as appendicitis.^8^ Evaluation for abdominal pain frequently necessitates time and resource-intensive diagnostics such as laboratory work or advanced imaging (ultrasound or computed tomography [CT]). Hoyt et al reported that APCs triage ED pediatric patients with abdominal pain with the same accuracy as physicians, but no study to date has investigated clinician category association with resource use in pediatric abdominal pain.^9^ In adult patients with abdominal pain, a large, multicenter cohort study showed no difference in diagnostic utilization by clinician category.^10^ Given this prior general ED data, we hypothesized that there would be no significant difference in LOS or resource utilization when comparing physicians with APCs.

## METHODS

### Study Design

We conducted a retrospective cohort study of pediatric patients who presented to a pediatric tertiary- care ED from May 2021–April 2022 with a complaint of abdominal pain. When possible, we followed tenets of optimal retrospective chart review as established by Worster et al, which are further delineated below.^11^ Data were acquired directly from the Cerner electronic health record (EHR) (Oracle Health, Kansas City, MO) through the hospital’s Clinical Data Warehouse (Worster element 9).^11^ As objective data was retrieved directly from the medical record, inter-observer reliability was not applicable (Worster element 7,8).^11^ The university’s institutional review board reviewed the study and deemed it exempt (Worster element 12).^11^

**Population Health Research Capsule**

What do we already know about this issue?

*Abdominal pain accounts for 10% of all pediatric ED visits. In adult abdominal pain there is no difference in testing between advanced practice clinicians (APC) and physicians.*

What was the research question?

*Is there a difference in length of stay or resource utilization when comparing physicians with APCs?*

What was the major finding of the study?

*APC encounters were 17 minutes longer than attending encounters (P <.005, 95% CI −29.9, −4.0) and 21 minutes longer than resident encounters (P <.001, 95% CI 11.4*–*30.6).*

How does this improve population health?

*Staffing practices by clinician group are another consideration for maximizing efficiency in pediatric emergency care and high-value care.*

### Study Setting and Population

The study sample included all ED encounters of patients 4–18 years of age with a chief complaint of abdominal pain (Worster element 2,10).^11^ We used this age cutoff to eliminate the infant and toddler patient groups that add additional surgical pathologies to consider, such as pyloric stenosis and intussusception. Subsequent visits for abdominal pain by the same patient were included as an independent encounter. While repeat visits may be more likely to result in higher resource use, this typically does not affect which clinician category sees the patient. We excluded those with the following medical comorbidities, conditions, or outcomes using International Classification of Diseases, 10^th^ Rev, (ICD-10) codes: non-infectious colitis including inflammatory bowel disease; pregnancy; g-tube presence; ventilator dependence; history of abdominal surgery and history of organ transplant, as well as any encounters that resulted in death (Worster element 2).^11^ These patients were excluded due to an increased likelihood that they would be evaluated by a physician-only team at the study site. Visits with Emergency Severity Index (ESI) levels 3, 4, and 5 were selected to best capture non-high acuity patients. This was done to minimize the potential for selection bias by clinician category, as there has been a reported tendency for APCs to avoid higher acuity cases in previous literature (Worster element 2).^11^,^12^

The study site had three clinical areas, and all areas had equal access to facility resources and diagnostic testing. Attendings primarily see patients on their own in the fast track area and occasionally in the teaching areas. The APCs would generally see patients independently with attending consultation available. For the first six months of employment, all APC patients are staffed with an attending prior to patient discharge. The APCs, residents, and attendings are present in the study ED 24 hours a day, seven days a week. The compensation model for attending physicians at the institution included both a group and individual relative value unit-based incentive plan. The APCs do not have a financial incentive related to patient volume, but their productivity is tracked and compared to national benchmarks.

The chief complaint on presentation was obtained from the EHR. This is selected from a drop-down menu by the triage nurse at the time of arrival to the ED and is based on the patient’s or family’s provided history. We excluded patients seen initially by students or independently by pediatric emergency medicine (EM) fellows moonlighting as general pediatric attendings, patients who left without being seen, or those who were transferred from another facility. Patients seen only by moonlighting pediatric EM fellows were excluded as they have varying levels of supervision depending on their year of training and individual comfort level.

### Study Protocol

We collected patient demographics and final ESI level for each encounter. For statistical reasons, ESI levels 4 and 5 were combined due to the low volume of ESI 5 visits. We determined ED clinician category determined by the physician or APC who signed the original note. When a patient was signed out, the clinician category was assigned based on the initial clinician. We defined emergency clinician by one of three categories. The first category was attending only, ie, encounters where the only clinician involved was either a physician board eligible in pediatric EM (PEM) or a general emergency pediatrician (GEP). The second category was APC, ie, encounters where the initial clinician was an APC. (Patients seen by NPs and PAs were combined into the APC clinician category as prior literature has shown no difference on the quality of care or admissions between these groups.^5^) Some APC visits had an attending evaluation when requested by the APC, typically for diagnostically challenging patients or before obtaining certain testing, such as CT). The third emergency clinician category was supervised resident, ie, encounters where the initial clinician was a resident physician. Supervised residents included those training in pediatrics, family medicine, EM, anesthesia, and transitional year interns. All supervised resident encounters included an evaluation by an attending physician before disposition. Some of these supervised resident encounters may have included an additional evaluation by a first- or second-year PEM fellow either separately or jointly with the attending, depending on attending comfort and fellow experience.

Approximately 20% of attending shifts have a dedicated fellow present. Encounters were considered supervised resident encounters for the <1% of visits where first- or second-year fellows saw patients initially and then staffed with an attending on a non-moonlighting shift. During the study period, there were 25 PAs, five NPs, 36 PEM attendings, and three GEPs who evaluated patients in the study ED. In our sample, data abstraction yielded all desired data apart from a few conflicting clinician categories, usually resulting from sign-out of the patient between categories of clinicians. These cases were all assigned to the original treating clinician category after manual chart review (Worster element 11).^11^

### Outcome Measures

The primary outcome measured was the ED LOS in minutes, obtained from a previously established hospital dashboard. The ED LOS is derived from the time of patient check-in to the ED until the electronic order completing the ED evaluation is placed (Worster element 3).^11^ Secondary outcome measures included the disposition of the patient (admitted or discharged), the rate of unplanned 72-hour return to the ED, and resource utilization metrics (Worster element 3).^11^ We studied patient disposition, as the decision to admit or discharge a patient can be taken independently by APCs and, thus, may vary between groups. We included as resource use metrics the presence or absence of lab studies, CTs, all other types of imaging studies (radiography, ultrasound, magnetic resonance imaging [hereafter referred to as “radiology studies”]), and specialist consultations. The decision to obtain a specific resource was considered more influential on the primary outcome of ED LOS than the total number of resources used.

Lab studies were limited to those from blood; we excluded urine and nasopharyngeal tests due to the relative ease of procurement. Use of CT was highlighted given the concern for radiation exposure in the pediatric population (Worster element 3).^11^ Final ICD-10 diagnosis codes were also collected for each encounter. Diagnostic groups were then created by one of the authors (NP), who consolidated related ICD-10 codes.

### Data Analysis

Data abstraction was completed by a systems analyst who was part of the hospital data warehouse group, using coded queries through SAP BusinessObjects (SAP SE, Walldorf, Germany), with output provided in Microsoft Excel (Microsoft Corporation, Redmond, WA). These abstractors are employees of the hospital who are trained in Cerner and SAP BusinessObjects and monitored by the hospital’s data warehouse management team (Worster element 1,5).^11^ The systems analyst was aware of the study objective but not the study hypothesis as part of the data request (Worster element 6).^11^

We used descriptive statistics, including proportions, medians, and interquartile ranges (IQR) to compare demographics between groups. Given non-normal distributions, the Kruskall-Wallis one-way analysis of variance was used to measure the difference between continuous variables. We assessed proportional differences in patient use metrics using Pearson chi-square tests. Multicollinearity was examined through a variance inflation factor cutoff of 2, and all other assumptions for linear and binominal logistic regression were met. Multivariate linear regression with estimated marginal means was employed to control covariates for ED LOS. Acknowledging the effect of skew on ED LOS data when comparing means instead of medians, we removed outliers, defined as values with *z*-scores ± 3.

When comparing attending visits and APC visits, attending visits were the reference group. For two-way comparisons including supervised resident encounters, resident visits were the reference group. For all resource utilization metrics, we performed binomial logistic regression first without covariates and then with covariates to obtain unadjusted and adjusted odds ratios (AOR). All statistical analyses were conducted in IBM SPSS Statistics 29 (IBM Corp, Armonk, NY), with *P* values < .05 considered statistically significant.

## RESULTS

### Characteristics of Study Subjects

**Figure 1:**
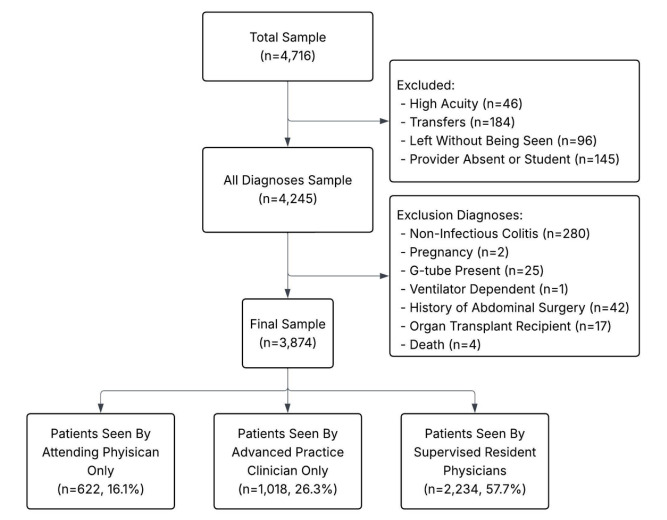
All patients 4–18 years of age who presented with a chief complaint of abdominal pain in the study period.

Of 4,716 eligible encounters during the study period, 3,874 (82%) met inclusion criteria (Figure 1). Of these visits, 622 (16%) were seen by an attending only, 1,018 (26%) by APCs, and 2,234 (58%) by supervised residents. Demographic and use characteristics of the sample are described in Table 1. The median ED LOS was 268 minutes; 831 (22%) encounters resulted in admission; and there were 170 (4%) 72-hour return visits. While 2,960 encounters (76%) had radiology studies and 2,228 (58%) had labs obtained, only 56 (1%) had CT.

### Main Results

We found a significant difference in ED LOS by clinician categories, although there was no significant difference in rates of admission or 72-hour return visits. All resource utilization metrics studied were significantly different between the groups including rate of obtaining lab tests, CT, consultations, and radiology studies (Table 2).

The APC encounters had a longer ED LOS (median 286 minutes, IQR 217–364) when compared to attending- only encounters (median 259 minutes, IQR 192–340) and supervised resident encounters (median 263 minutes, IQR 194–340). These differences remained significant when comparing mean ED LOS among groups and controlling for the covariates of age, sex, race, and acuity. (Table 3)

**Figure 2:**
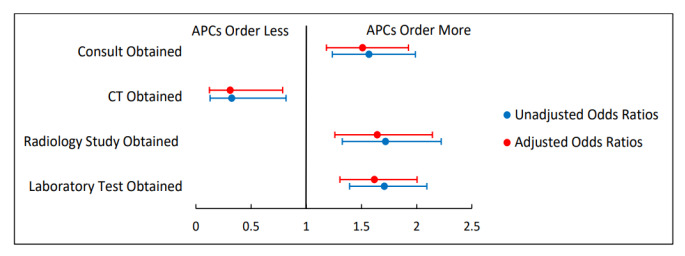
Rates of resource utilization of emergency department encounters for abdominal pain seen by attendings compared to those seen by advanced practice clinicians. Adjusted odds ratios control for the covariates of age, sex, race, and acuity level. *APC*, advanced practice clinician*; CT*, computed tomography; *ED*, emergency department.

**Figure 3:**
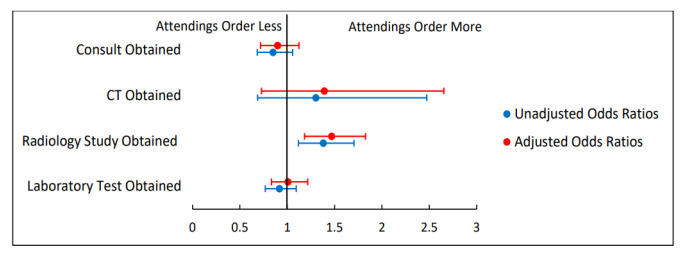
Rates of resource utilization of emergency department encounters for abdominal pain seen by supervised residents compared to those seen by attendings. Adjusted odds ratios control for the covariates of age, sex, race, and acuity level. *CT*, computed tomography; *ED*, emergency department.

**Figure 4:**
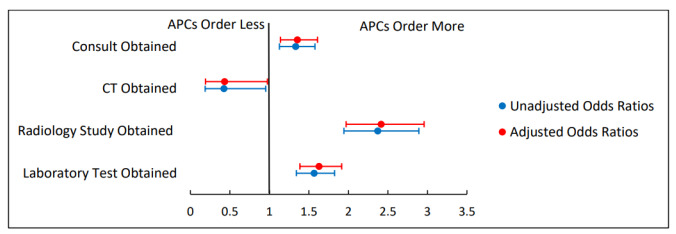
Rates of resource utilization of emergency department encounters for abdominal pain seen by supervised residents compared to those seen by advanced practice clinicians. Adjusted odds ratios control for the covariates of age, sex, race ,and acuity level. *APC*, advanced practice clinician*; CT*, computed tomography; *ED*, emergency department.

Figures 2–4 present unadjusted and adjusted ORs calculated when comparing rates of resource utilization among attending only, supervised resident, and APC encounters. When compared to attending encounters, APC encounters were more likely to obtain a consult (aOR 1.51, 95% CI 1.18–1.93); less likely to obtain a CT (aOR 0.31, 95% CI 0.12–0.79); more likely to obtain a radiology study (aOR 1.64, 95% CI 1.26–2.14); and more likely to obtain a lab test (aOR 1.62, 95% CI 1.30–2.00) (Figure 2). The only significant difference when comparing attending only encounters to supervised resident encounters was that an attending-only encounter was more likely to include a radiology study (aOR 1.47, 95% CI 1.18–1.83) (Figure 3). Comparing APC encounters with supervised resident encounters showed that APC encounters were more likely to include a consult (aOR 1.35, 95% CI 1.14–1.61); less likely to obtain a CT (aOR 0.43, 95% CI 0.19–0.98); more likely to obtain a radiology study (aOR 2.41, 95% CI 1.97–2.96); and more likely to obtain a lab test (aOR 1.63, 95% CI 1.39–1.92) (Figure 4).

When comparing subspecialty consults, lab tests, and radiology studies, analysis was controlled for age, sex, race, and acuity. When analyzing rates of obtaining CT, acuity was removed as a covariate due to low counts (one CT obtained in acuity level 4+5).

### Analysis by Diagnostic Groups

The most common ICD-10 diagnosis was unspecified abdominal pain (R10.9) accounting for 617 (15.9%) of the sample population. Table 4 shows the five most common diagnostic groupings. Attendings had a higher relative proportion of visits for constipation (14.5% of all attending visits, vs 10.1% of all APC visits, *P* = .02); and APCs had a higher relative proportion of encounters for appendicitis (7.9% of all APC visits vs 5.6% of all attending visits, *P* = .04).

**Figure 5:**
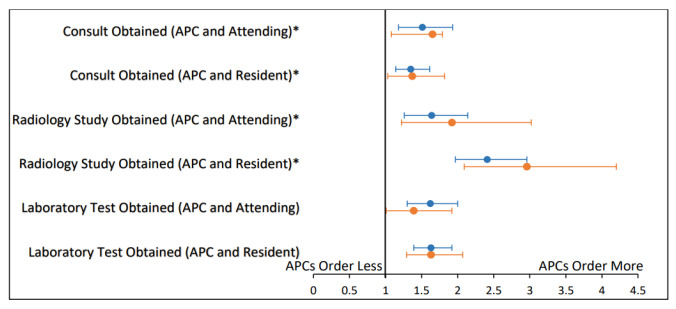
Comparing rates of obtaining consults, radiology studies, and lab orders between clinician categories in the total sample and the unspecified abdominal pain sub-group. *Indicates that the previously seen association in the total sample became stronger within the unspecified abdominal pain sub-group. *aOR*, adjusted odds ratio; *APC*, advanced practice clinician; *CT*, computed tomography.

Resource utilization comparisons were repeated for the unspecified abdominal pain diagnostic group and compared to the total sample (Figure 5). For consults, the adjusted OR was higher in the unspecified abdominal pain group than the total sample when comparing APCs and attendings (aOR 1.65, 95% CI 1.08–1.79, vs aOR 1.51, 95% CI 1.18–1.93) and when comparing APCs and residents (aOR 1.37, 95% CI 1.03–1.82, vs aOR 1.35, 95% CI 1.14–1.61). For obtaining a radiology study this was also true when comparing APCs and attendings (aOR 1.92, 95% CI 1.22–3.02, vs aOR 1.64, 95% CI 1.26–2.14) and when comparing APCs and residents (aOR 2.96, 95% CI 2.09–4.2, vs aOR 2.41, 95% CI 1.97–2.96). For obtaining a lab test, the adjusted OR was lower for the unspecified abdominal pain group as compared to the total sample when comparing APCs and attendings (aOR 1.39, 95% CI 1.01–1.92, vs aOR 1.62, 95% CI 1.30–2.00) and equivalent when comparing APCs and residents (aOR 1.63, 95% CI 1.29–2.07, vs aOR 1.63, 95% CI 1.39–1.92). We did not compare use of CT due to the low number done in the unspecified abdominal pain group (n = 12).

## DISCUSSION

This single-center study compared clinician categories based on their LOS and rates of resource utilization in the evaluation of pediatric ED encounters for complaints of low-acuity and low-complexity abdominal pain. Abdominal pain can be difficult to diagnose but is a common chief complaint, encompassing a wide range of potential etiologies and outcomes. The potential diagnostic uncertainty of pediatric abdominal pain resulted in 1,711 (44%) patient encounters in our sample being given a final diagnosis of “unspecified abdominal pain,” by far the most common final diagnosis group. Adding to the difficulty of evaluating this chief complaint, both benign and dangerous etiologies were frequent, as both constipation (second most common, n = 444, 11.5%) and appendicitis (fourth most common, n = 241, 6.2%) were among the top five diagnosis groups. There were significant differences in the proportions of constipation and appendicitis seen by each clinician category. To investigate the effect of this difference on our results, all analyses were repeated for the sample, first excluding patients with constipation and next excluding patients with appendicitis. In the repeat analyses, all results retained the original directionality and remained statistically significant.

Compared to encounters conducted by physicians, those conducted by APCs as the primary clinician had statistically significant longer visits. The average APC encounter was 8.7% longer than the average supervised resident encounter, and 10.4% longer than the average attending-only visit. The clinical significance of this difference in throughput becomes more apparent when considering the frequency of abdominal pain as a chief complaint. Abdominal pain accounts for approximately 10% of all pediatric ED visits.^8^ Given that our average daily census for the study period was 230 encounters, this would equate to 23 visits for abdominal pain and a 391-minute (APC vs attending) to 483-minute (APC vs resident) difference in LOS per day. We believe that this would be a meaningful clinical difference when evaluating ED LOS metrics.

Most APC encounters in the study ED are seen independently, whereas all resident encounters are discussed with an attending who then also personally evaluates the patient. Attending-only encounters often occur concurrently with resident supervision. If these findings are reproduced more broadly, it would indicate an important consideration when staffing additional APCs in lieu of physicians: labor costs may be lower, but clinical care may be less efficient and more costly, potentially offsetting savings in salary. Our findings were for low-acuity and low-complexity patients; if reproduced across larger samples and different centers, this would suggest a reassessment of staffing “fast track” areas with APCs, as 86% of academic EDs do.^13^ Some of the difference in LOS is likely associated with the higher rates of resource utilization for these encounters by APCs, as APCs were more likely to obtain consults, radiology studies, and lab studies than those with supervised residents or those with attending physicians only.

Data on throughput differences by clinician group in adult EDs remain mixed. A multicenter study across 94 EDs involving 13 million visits found that APCs saw patients at half the rate of physicians.^5^ In contrast, a national survey of 95,000 visits reported shorter LOS for patients seen by APCs.^14^ Regarding resource utilization, findings are similarly variable. One ED-level study reported higher admission and imaging rates in EDs with APC coverage.^15^ However, broader literature suggests that independently practicing APCs may use fewer diagnostic tests.^14^,^16^ When focusing specifically on adult abdominal pain visits, Pines et al found no significant differences in lab or imaging use between clinician groups.^10^

In pediatric EDs, existing literature has primarily focused on respiratory and low-acuity complaints. A 2021 study on bronchiolitis found that NPs were less likely to order tests or treatments when compared to physicians.^7^ However, the study did not adjust for illness severity, and the large difference in admission rates (2% for NP patients vs 31% for physician patients) indicates this may have influenced the results. A 2012 two-site study of 150,000 low-acuity encounters found no differences in testing by clinician type.^17^ More recently, a single-center study (2017–2020) reported longer LOS for PAs compared to pediatricians in low-acuity cases.^6^ Although sensitivity analyses by chief complaint were conducted, results were only reported on respiratory complaints.

When comparing throughput and resource utilization between physician groups, there was little difference between attending-only encounters and those including supervised residents. This is consistent with prior general ED data from teaching hospitals.^18^ This was an expected finding in our cohort, given that low- acuity patients seen in the study ED are typically evaluated by a resident and discussed with the attending prior to a care plan being enacted.

Interestingly, the data revealed that at our site, attendings were more likely to order a radiology study when conducting a visit alone compared to visits with a resident. This may be due to time constraints faced by the attendings, as most attendings who see patients on their own do so while also supervising residents. Reassurance is a core aspect of pediatric medicine but can be time intensive and emotionally consuming.^19^ When deliberating between a lengthy discussion about the utility of a radiographic test or simply ordering it, time constraints may outweigh an attempt at reassurance or shared decision-making. This principle is likely more applicable for radiography studies since no pain is caused to the patient (compared to lab testing) and there is often little (radiograph) to no (ultrasound) radiation to the patient. Adequate reassurance is also easier to provide when sharing the encounter with a resident, who can provide repetition and spend more time with the patient and family. This efficiency of multiple clinicians may explain why there was no difference in ED LOS between attending and resident encounters despite the additional requirement of staffing patients for resident visits.

In contrast to the other resources studied, our results showed that visits with APCs were less likely to include CT when compared to either physician group. This could be a balancing outcome of the higher number of labs and consults obtained by APCs, allowing them to correctly triage the need for a CT. This is supported by APCs not having a higher rate of 72-hour ED return visits compared to the attending-only or supervised resident groups. Another potential explanation is the cultural feature of our site where most APCs would discuss a patient with a physician prior to ordering a CT, adding another step of deliberation.

We conducted further analysis of our two most common diagnosis groups (unspecified abdominal pain and constipation) to identify themes to maximize efficient and high-value care of pediatric patients with abdominal pain. These analyses revealed two areas for investigation: variations in clinician comfort with diagnostic uncertainty; and a recommitment to high-value care. Comfort with uncertainty applies to encounters with a final diagnosis of unspecified abdominal pain, which is used when no other etiology is found. When considering encounters with a final diagnosis of unspecified abdominal pain, differences in resource utilization between APCs and physicians were stronger than in the overall data set for obtaining both consults and radiology studies (Figure 5). The effect of varying comfort with diagnostic uncertainty becomes more convincing when considering prior work in bronchiolitis. When evaluating bronchiolitis in the pediatric ED, a condition with less diagnostic uncertainty than abdominal pain, APCs were shown to use fewer resources compared to physicians.^7^

Although diagnostic uncertainty can be uncomfortable for any clinician, the results in Figure 5 indicate stress from uncertainty may be associated with ordering practices in APC encounters more than physician encounters at our center. This finding is consistent with adult ED research that showed no association between imaging orders for abdominal pain and physician stress with uncertainty,^20^ while Navandan et al found that APCs had higher anxiety due to uncertainty scores, a higher reluctance to disclose uncertainty, and used more resources to evaluate pediatric respiratory complaints.^21^ Formal didactics around uncertainty in medicine are being introduced for medical students,^22^ and tools such as an uncertainty communication checklist^23^ could be a valuable addition to the curriculum for APCs practicing in the high-uncertainty environment of the ED.

When investigating encounters with a final diagnosis of constipation, there was no significant difference in median LOS (*P* = .23) or any of the utilization metrics between clinician categories. Our rates of imaging in encounters resulting in a diagnosis of constipation were high (80.6%), regardless of the clinician category. Imaging in pediatric constipation is associated with a higher incidence of misdiagnosis^24^ and is not recommended by the American Academy of Pediatrics as part of the ”Choosing Wisely” campaign.^25^,^26^ Certain encounters likely require some diagnostic evaluation before constipation can be chosen as the final diagnosis, and rates of imaging are higher in the ED setting (70%) compared to outpatient clinics (5%).^27^ Encouragingly, our findings represent an improvement from rates obtained at the same hospital 11 years prior (80.6% vs 92.1%, *P* < .001). However, further improvement could come from the introduction of an educational module, such as the one Kurowski and colleagues implemented in a similar site that successfully reduced imaging when making the diagnosis of constipation.^28^ Given that our 72-hour return rate of 4% is still within the previously reported range for pediatric EDs (2.1%–5.2%)^29^ despite our higher rates of imaging, it is unlikely that the additional imaging is preventing missed diagnoses.

## LIMITATIONS

There were several limitations to our study. As a single-center study, although clinician categories were not small, some of the differences found may be more related to individual-level characteristics as opposed to professional background. However, testing this assumption on average ED LOS did show that neither the APC nor the attending-only group had any outliers with a *z*-score outside ±3. Unsurprising given the more than eight residency programs that help staff the ED, the supervised resident group did have nine outliers outside a *z*-score of ±3. The exclusion of these outliers changed the median ED LOS for the supervised resident group by less than one minute.

A related limitation to be incorporated in future studies is that years of experience were not considered, which may^30^ or may not^31^ be associated with tolerance for uncertainty. Another aspect that could contribute to throughput and resource utilization that was not considered here was the time of day at presentation. While all three clinician groups provide continuous coverage, the staff-to-patient ratio decreases overnight. This includes clinicians as well as ancillary staff such as radiology techs and lab techs. This may influence the selection and turnaround time of laboratory and radiology testing. Attendings, supervised residents, and APCs all staff the ED 24/7, which should mitigate this potential effect when comparing among clinician categories.

Another limitation of the study was that the results of lab testing and imaging studies were not studied. While this would be especially informative when assessing the value of these studies, it was outside the scope of this study. Additionally, we attempted to control for medical complexity by eliminating patient encounters with certain comorbidities thought to influence patient selection; however, this was not an exhaustive list. Additionally, ESI is an imperfect tool to capture patient acuity and reflects anticipated resource utilization. While it successfully limited high-acuity patients (< 1% admitted to the intensive care unit), only the final ESI level was collected, which could have been changed during the encounter to reflect estimated resource use. Our CT-related findings should also be considered in the context of a low sample size, as only 1.4% of encounters had a CT performed.

Finally, some APC encounters included an attending physician evaluation by APC request based on site-specific culture. In our dataset, there was no reliable way to delineate which APC encounters included discussion with an attending physician, potentially before diagnostic tests such as CT were ordered, although this would be uncommon. An APC request for additional review by the attending physician may also have contributed to some increase in length of stay. We were also unable to definitively attribute testing to the correct clinician category if sign-out occurred during the encounter. In the study site, most sign-outs occur between like clinician categories.

## CONCLUSION

In this single-center, retrospective study, we found an association between clinician category and ED throughput and resource utilization in low-acuity pediatric patients presenting with a chief complaint of abdominal pain. The encounters staffed by advanced practice clinicians were on average 17 minutes longer than attending encounters and 21 minutes longer than supervised resident encounters. They were also more likely to include lab testing, radiology studies, and consults than resident or attending-only encounters. Further multicenter research may minimize the effects of individual-level characteristics, and educational efforts across all levels of medical professionals can promote adherence to evidenced-based care. These interventions may reduce unnecessary imaging and testing for common pediatric conditions such as constipation, thereby optimizing throughput and resource utilization.

## Figures and Tables

**Table 1:** Demographic and visit characteristics of ED patients presenting with abdominal pain by clinician category.

Sample Size	Total N=3,874 (100%)	Attending Only n=622, (16%)	APC n=1,018 (26%)	Supervised Resident n=2,234 (58%)	P-value
Age, median (IQR), Years	11 (7–15)	10 (7–14)	11 (7–14)	11 (7–15)	**<.005** *
Female Sex, n (%)	2,351 (61)	345 (56)	634 (62)	1,372 (61)	**.01** *
Race, n (%)					.63
White	2,875 (74)	459 (74)	767 (75)	1,649 (74)	
Black	706 (18)	108 (17)	185 (18)	413 (19)	
Asian	176 (5)	34 (6)	40 (4)	102 (5)	
Other	117 (3)	21 (3)	26 (3)	70 (3)	
ESI Level, n (%)					.07
3	3,321 (86)	515 (83)	891 (88)	1,915 (86)	
4	527 (14)	100 (16)	120 (12)	307 (14)	
5	26 (1)	7 (1)	7 (1)	12 (1)	

* Indicates statistical significance at *P* < .05,

*APC*, advanced practice clinician; *ED*, emergency department; *ESI*, Emergency Severity Index; *IQR*, interquartile range.

**Table 2:** Throughput, disposition, and resource use of emergency department patients presenting with abdominal pain by clinician category.

Sample Size	Total N=3,874 (100%)	Attending Only n=622 (16%)	APC n=1,018 (26%)	Supervised Resident n=2,234 (58%)	P-value
ED LOS median (IQR), minutes	268 (199, 348)	259 (192,340)	286 (217, 364)	263 (194, 340)	**<.001** **
Return visit, n (%)	170 (4)	30 (5)	41 (4)	99 (4)	.61
Admitted, n (%)	831 (22)	128 (21)	226 (22)	477 (21)	.63
Laboratory test obtained, n (%)	2,228 (58)	329 (53)	669 (66)	1,230 (55)	**<.001** **
CT obtained, n (%)	56 (1)	13 (2)	7 (1)	36 (2)	**.04** *
Consult obtained, n (%)	937 (24)	127 (20)	292 (29)	518 (23)	**<.001** **
Radiology study obtained, n (%)	2,960 (76)	484 (78)	873 (86)	1,603 (72)	**<.001** **

* Indicates significance at *P* <.05.

** indicates significance at *P* < .001.

*APC*, advanced practice clinician; *CT*, computed tomography; *ED*, emergency department; *IQR*, interquartile range; *LOS*, length of stay.

**Table 3:** Pairwise comparisons of mean emergency department length of stay in minutes by clinician category.

Unadjusted comparisons+	ED LOS (Minutes)			Adjusted comparisons+∇	ED LOS (Minutes)		
	
	Mean difference	P-value	95% CI		Mean difference	P-value	95% CI
Attending vs APC	−22.8	<.001**	(−36.0, −9.6)	Attending vs APC	−16.9	< .005*	(−29.9, −4.0)
Attending vs Supervised Resident	−1.5	.96	(−13.1, 10.4)	Attending vs Supervised Resident	4.1	1.0	(−7.5, 15.6)
APC vs Supervised Resident	21.5	<.001**	(11.4, 31.5)	APC vs Supervised Resident	21	<.001**	(11.4 30.6)

+ Last group is reference group.

∇ Controlling for age. sex, race and acuity.

* Indicates significance at *P* < .05.

** Indicates significance at *P* < .001.

*APC*, advanced practice clinician; *ED*, emergency department; *LOS*, length of stay.

**Table 4:** Five most common diagnostic groupings by final *International Classification of Diseases, 10^th^ Rev*, code in emergency department patients presenting for abdominal pain.

Diagnostic Group	Included ICD-10 Codes	Number of Encounters	Percentage of Sample	P-value
Unspecified Abdominal Pain	R10.9, R10.31, R10.33, R10.13, R10.32, R10.11, R10.30, R10.84, R10.12, R10.10, R10.813	1,711	44.2%	.27
Constipation	K59.00, K59.09, K59.04,	444	11.5%	**.02** *
Viral Infections	A08.4, B34.9, U07.1, J06.9, B27.9, A09, B19.9, B27.00, B30.8	258	6.7%	.15
Appendicitis	K35.8, K35.32, K35.3,K35.33, K35.890, K35.2, K35.21, K35.891	241	6.2%	**.04** *
Vomiting Unspecified	R11.10, R11.2, R11.0, R11.14	165	4.3%	.11

* Indicates statistical significance at *P* < .05.

*ED*, emergency department; *ICD-10, International Classification of Diseases, 10^th^ Rev.*
